# Ranges of Injury Risk Associated with Impact from Unmanned Aircraft Systems

**DOI:** 10.1007/s10439-017-1921-6

**Published:** 2017-09-14

**Authors:** Eamon T. Campolettano, Megan L. Bland, Ryan A. Gellner, David W. Sproule, Bethany Rowson, Abigail M. Tyson, Stefan M. Duma, Steven Rowson

**Affiliations:** Virginia Polytechnic Institute and State University, Blacksburg, VA USA

**Keywords:** Drone, Skull, Brain, Concussion, Cervical spine, Neck

## Abstract

Regulations have allowed for increased unmanned aircraft systems (UAS) operations over the last decade, yet operations over people are still not permitted. The objective of this study was to estimate the range of injury risks to humans due to UAS impact. Three commercially-available UAS models that varied in mass (1.2–11 kg) were evaluated to estimate the range of risk associated with UAS-human interaction. Live flight and falling impact tests were conducted using an instrumented Hybrid III test dummy. On average, live flight tests were observed to be less severe than falling impact tests. The maximum risk of AIS 3+ injury associated with live flight tests was 11.6%, while several falling impact tests estimated risks exceeding 50%. Risk of injury was observed to increase with increasing UAS mass, and the larger models tested are not safe for operations over people in their current form. However, there is likely a subset of smaller UAS models that are safe to operate over people. Further, designs which redirect the UAS away from the head or deform upon impact transfer less energy and generate lower risk. These data represent a necessary impact testing foundation for future UAS regulations on operations over people.

## Introduction

Small unmanned aircraft systems (UAS) represent a potentially substantial market as their use becomes more commonplace. It has been estimated that the economic benefit from UAS operations may exceed $82.1 billion by 2025.^2^,^26^ Since 2008, the Federal Aviation Administration (FAA) has been attempting to incorporate the use of UAS within the national airspace system (NAS).^1^,^6^,^9^,^16^,^21^,^25^ The FAA Modernization and Reform Act of 2012 set forth directives towards assessing the risks associated with operational UAS.^26^ Part 107 of Title 14 Code of Federal Regulations, which stipulates the regulations regarding UAS flight, was signed into effect in 2016. Operational specifications limit the mass of any UAS to 55 lbs. (25 kg), maximum speed to 100 mph (45 m/s), and maximum altitude to 400 ft. (122 m) above ground level. The rule further states that all UAS must be operated within visual line-of-sight of the pilot and may not operate over persons.^9^

Unmanned aircraft systems applications are currently limited to monitoring and inspection for agriculture, power lines, and bridges, educational pursuits, research and development, aerial photography, and rescue operations.^9^ Two applications considered to be among the largest potential markets for UAS, freight transport and public safety applications by police officers or firefighters, are not included in this list.^2^,^5^ These operations would require flight over people, which the FAA has yet to allow for two major reasons: a paucity of safety data available for risk to humans and that no other country with UAS regulations allows for operation over people.^8^,^9^,^16^

Safety standards exist in most industries to regulate the potential for catastrophic injury and death. Of note, current safety standards in the automotive and sport industries have been very effective in limiting catastrophic and fatal events. In the automotive industry, Federal Motor Vehicle Safety Standards (FMVSS) 208 and 214 specify minimum occupant protection requirements for frontal and side impact motor vehicle crashes. These two standards, combined with the New Car Assessment Program (NCAP), which provides consumers with ratings of occupant protection by vehicle model beyond the standards, have reduced the fatality rate associated with motor vehicle crashes by 80% over the last 50 years.^15^ In the sport industry, the National Operating Committee on Standards for Athletic Equipment (NOCSAE) governs standards that specify minimum performance requirements for protective headgear. When the NOCSAE standard for football helmets was first implemented, the number of fatal head injuries in football was reduced by 74%.^13^ Safety standards such as these have been so effective because they limit loads transferred to the body during impact events.

Impact safety standards employ pass-fail thresholds for biomechanical parameters experienced by a human surrogate. In order to be certified as safe, meaning use of the product is unlikely to result in catastrophic or fatal injury, impact tests of products must produce biomechanical parameters below the threshold. It is important to note that these thresholds represent a specified risk of catastrophic or fatal outcome that is considered acceptable. Passing the standards does not imply that products are injury-proof. People still die in car crashes and football players still occasionally die due to head injury. Rather, the likelihood of these outcomes are minimized by regulating impact performance.

With the economic and public benefits spurring UAS regulations towards more applications and eventually flight over people, there is a need to understand and limit the risk to human life in the event of UAS failure in the air. The objective of this study was to estimate the range of head and neck injury risk to humans due to UAS malfunction by conducting live flight and falling impact tests with a range of commercially-available UAS. This testing represents a first step towards developing a UAS safety standard that minimizes threat to human life.

## Materials and Methods

**Figure 1:**
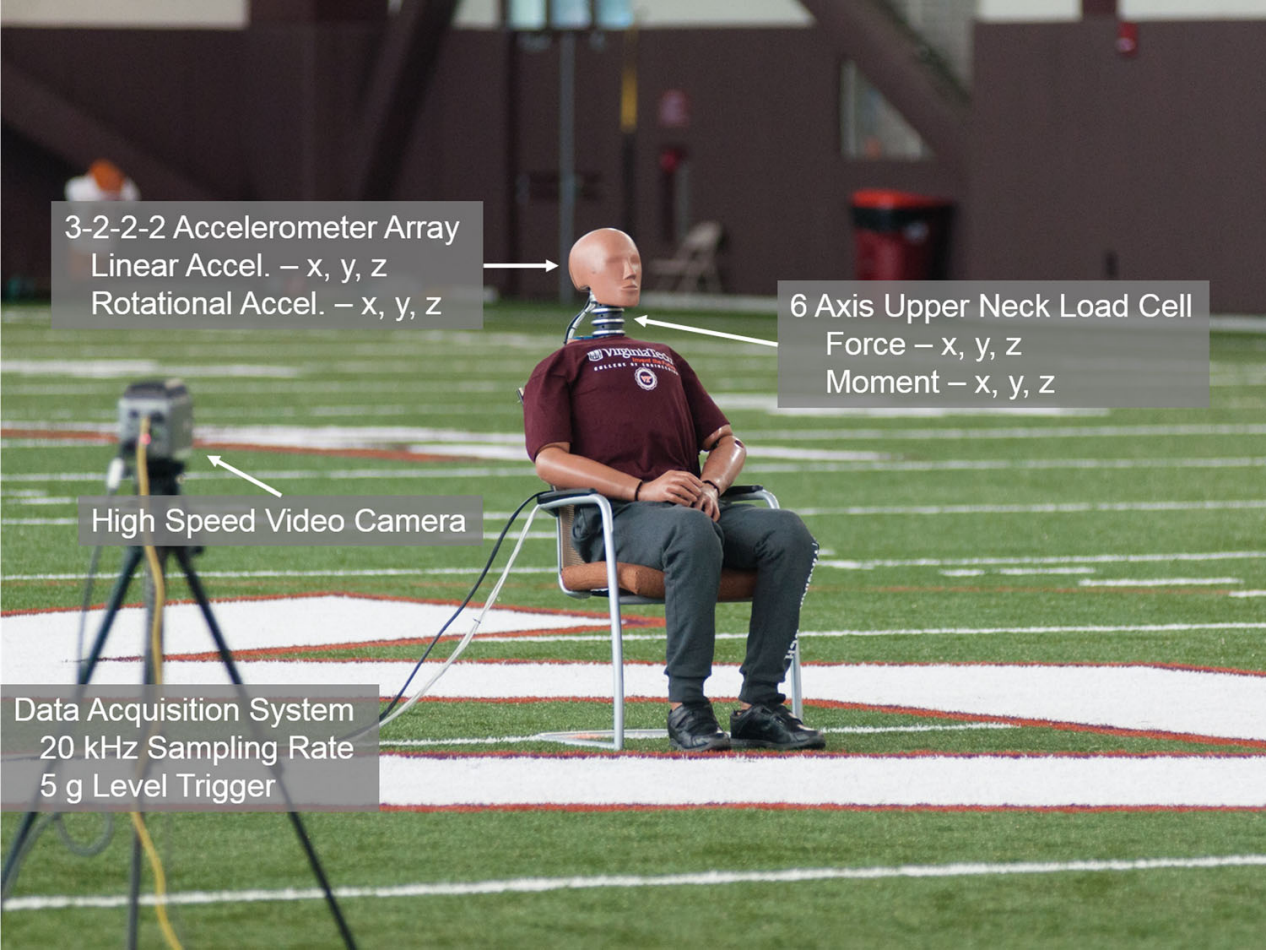
Experimental set-up for live flight tests. Head accelerations and neck loads were measured during impacts to relate to risk of serious head and neck injury. A similar experimental set-up was utilized for the falling impact tests.

Three commercially-available UAS were tested in this study. The UAS varied in mass and maximum speed in order to assess a range of potential energy inputs (Table 1). It should be noted that all of these UAS fall within the mass and speed limits regulated by the FAA.^9^ The UAS were tested by performing two kind of tests: flight tests and falling impact tests. In the flight tests, an operational UAS was flown into the head of the Hybrid III test dummy while a non-operational UAS was dropped from a height of 5.5 m onto the head of the Hybrid III test dummy for the falling impact tests. Flight tests were conducted in an indoor testing facility that measured 120 × 55 × 18 m (Fig. 1). This large space represented an open and controlled testing environment to conduct these live flight scenarios. Each UAS was accelerated over a distance of about 40 m in an effort to impact the Hybrid III head at full speed. Falling impact tests were conducted in a dedicated drop space with a high ceiling and an upper level from which to safely drop the UAS and achieve an impact velocity of 10 m/s. Batteries were removed from the UAS for these falling impact tests and replaced with an equivalent mass in order to minimize risk of fire during the testing process. The destruction of the UAS upon impacting the Hybrid III or ground during testing limited the number of tests that could be completed. Between 1 and 3 flight impact tests were conducted for each UAS by a certified pilot (Table 1). Falling impact tests were repeated between 5 and 7 times for each UAS model since safe operation was no longer a limitation. A variety of impact orientations were able to be tested to assess the spectrum of potential impact configurations and their corresponding risk.

**Table 1:** Summary of UAS models and test matrix.

UAS model	Mass (kg)	Speed (m/s)	Live flight tests (#)	Falling impact tests (#)
DJI phantom 3	1.2	16	3	7
DJI inspire 1	3.1	22	2	6
DJI S1000+	11	18	1	5

All three models were well below the maximum mass and speed thresholds currently allowed by the FAA. The overall design also differed between models, allowing for evaluation of impact energy transfer for different UAS components. More falling impact tests were conducted than live flight tests. Unmanned aircraft systems were tested until the structure was compromised beyond repair. A variety of impact orientations were evaluated during the falling impact phase of testing

All tests were conducted with an instrumented 50th percentile Hybrid III test dummy. The Hybrid III head was instrumented with a nine accelerometer array (7264-2000b, Endevco, San Juan Capistrano, CA).^17^ Three groups of 2 single-axis accelerometers were orthogonally mounted to the skull and 3 single-axis accelerometers were positioned at the center of gravity (CG) of the head. This orientation allowed for determination of linear and rotational accelerations about the CG of the head. The neck of the Hybrid III dummy was instrumented with a six-axis upper neck load cell (Denton 1716, Rochester, MI) to measure forces and moments about the *x*, *y*, and *z* axes. All data were sampled at 20 kHz (TDAS SLICE PRO SIM, DTS, Seal Beach, CA) with a 5 g level trigger along the axis of impact. A high speed video camera (Phantom v9, Vision Research, Wayne, NJ) sampling at 500 frames per second was used to determine the UAS orientation at impact.

A four-pole phaseless Butterworth low-pass filter was applied with a channel frequency class (CFC) 1000 for force data and linear acceleration data taken from the three accelerometers located at the CG of the head and a CFC 600 for moment data. Acceleration data were filtered at CFC 180 to compute rotational accelerations.^17^

The measured resultant head accelerations and the neck forces and moments were used to assess risk of catastrophic or fatal injury to a human. Risk of skull fracture was determined by using the head injury criterion (HIC) value from each impact (Eq. 1).^10^,^11^,^20^ HIC is calculated for time durations lasting a maximum of 15 ms. A HIC value of less than 700 is currently required by FMVSS and NCAP during automotive impact tests to pass the safety standard.

1 $$HIC = \left\{ {(t_{2} - t_{1} )\left[ {\frac{1}{{t_{2} - t_{1} }}\int\limits_{{t_{1} }}^{{t_{2} }} {a(t)dt} } \right]^{2.5} } \right\}_{\hbox{max} }$$

where *a* is acceleration and *t* is time. The potential for risk of concussion was estimated using a risk function that considers both linear and rotational resultant head acceleration.^22^,^23^

FMVSS and NCAP consider peak axial force and the Neck Injury Criteria (*N*_ij_) when assessing neck injury risk.^14^,^19^ *N*_ij_ considers 4 different loading modes: tension-extension, tension-flexion, compression-extension, and compression-flexion. A *N*_ij_ value less than 1 is required by FMVSS and NCAP to pass the safety standard.

2 $$N_{\text{ij}} = \frac{{F_{\text{z}} }}{{F_{\text{int}} }} + \frac{{M_{\text{y}} }}{{M_{\text{int}} }}$$

where *F*_z_ is the axial neck force, *F*_int_ is the critical neck load for the loading condition (i.e., compression or tension), *M*_y_ is the neck bending moment, and *M*_int_ is the critical bending moment for the loading condition (i.e., flexion or extension). The critical neck load does not vary between tension and compression and is 4500 N. The critical bending moment is dependent on the loading condition and is 310 N-m in flexion and 125 N-m in extension.

The head and neck injury risk functions employed in this study consider the risk of Abbreviated Injury Scale (AIS) 3+ injuries.^3^ The AIS is an injury coding system used to classify injury severity. The AIS classifies injuries from 1 to 6, where AIS 1 is minor, AIS 2 is moderate, AIS 3 is serious, AIS 4 is severe, AIS 5 is critical, and AIS 6 is maximum (not survivable).

## Results

### Flight Tests

**Figure 2:**
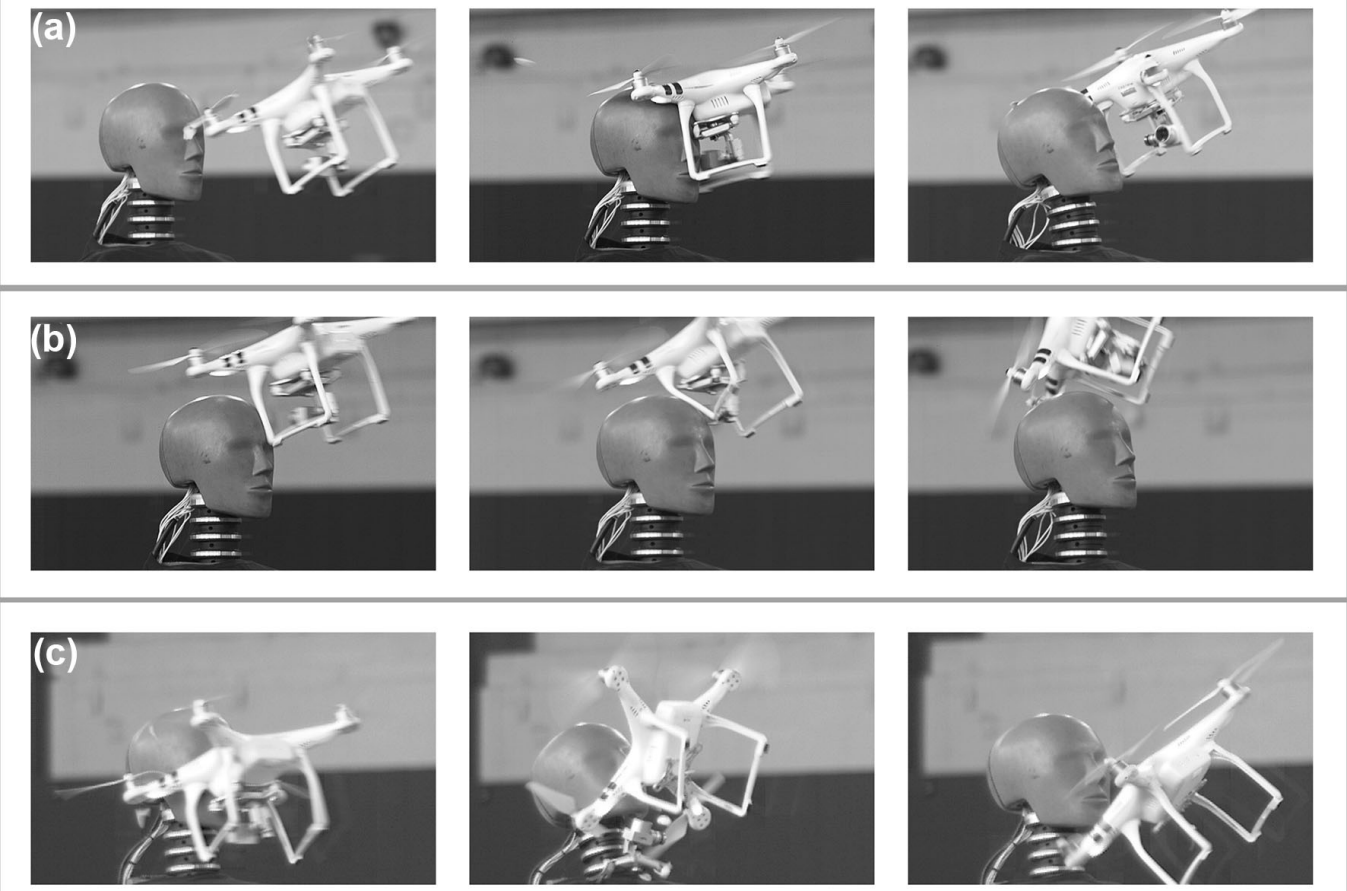
Live flight tests with DJI Phantom 3. (a) Even though the center of mass of UAS struck the Hybrid III head, the propeller struck first, limiting the energy transfer to the head; (b) One of the rubber legs of the UAS struck the Hybrid III head before deforming and rebounding away. Limited energy transfer was achieved for this live flight test; (c) The center of mass of the UAS directly struck the Hybrid III head in the face. This test likely approached maximal energy transfer from this model UAS to the head.

Three successful flight impact tests were conducted with the DJI Phantom 3. In the first flight, the left-front propeller blade struck the face first, causing the UAS to rotate (Fig. 2a). Then, the mass of the UAS itself struck the Hybrid III head above the left eye. Linear peak resultant acceleration measured 52 g and rotational peak resultant acceleration measured 4701 rad/s^2^. In the second flight, one of the UAS legs struck the top of the head of the Hybrid III and then deformed, causing the UAS to rotate away from the head, end-over-end (Fig. 2b). This limited energy transfer resulted in a peak linear acceleration of only 7.2 g and a peak rotational acceleration of 878 rad/s^2^. In the last flight, the center of mass of the UAS impacted the Hybrid III directly in the face, with components breaking off upon impact (Fig. 2c). Peak accelerations of 72 g and 4689 rad/s^2^ were measured. These three tests represented distinct impact orientations that led to different biomechanical outcomes, albeit with relatively low risk overall (Table 2). The low mass of the DJI Phantom 3 resulted in low levels of neck loading during these live flight tests.

**Table 2:** Biomechanical summary of live flight impacts.

UAS model	Phantom 3			S1000+
Trial	Flight A	Flight B	Flight C	Flight A
Peak linear acceleration (g)	52	7.2	72	43
Peak rotational acceleration (rad/s^2^)	4701	878	4689	2097
Peak axial force (*N*)	234	99	696	285
HIC_15_	30	1	59	12
*N*_ij_	0.07	0.03	0.61	0.43

For the DJI Phantom 3, impact severity varied with UAS orientation and was greatest (Flight C) when the center of mass striking the Hybrid III head was aligned during impact. Despite the greater mass of the S1000+ relative to the Phantom 3, impact severity was lower for this flight test

**Figure 3:**
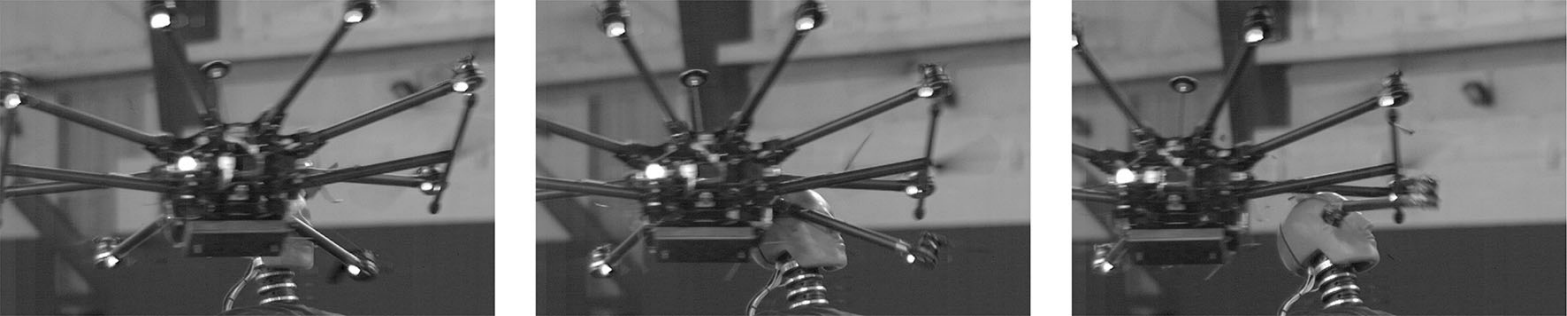
Live flight of DJI S1000+. One of the eight propeller arms struck the front of the Hybrid III head before deforming upon impact. The rest of the UAS continued forward, resulting in separation between the impacting propeller arm and the UAS.

No live flight tests with the DJI Inspire 1 proved to be successful. One attempt struck the Hybrid III in the abdomen before flying off, while the final attempt struck the shoulder and led to damage that could not be repaired conveniently. A single successful flight test was conducted with the DJI S1000+. One of the eight propeller arms struck the Hybrid III in the face before breaking off while the rest of the UAS continued its flight away from the test dummy (Fig. 3). Peak accelerations were measured to be 43 g and 2097 rad/s^2^. Though the mass of the DJI S1000+ was the greatest among all those tested, the center of mass rotated around the head and was not aligned during impact, leading to lower measured biomechanical values (Table 2). A portion of a propeller blade from the arm that struck the face broke off and became lodged in the Hybrid III’s skin.

### Falling Impact Tests

**Figure 4:**
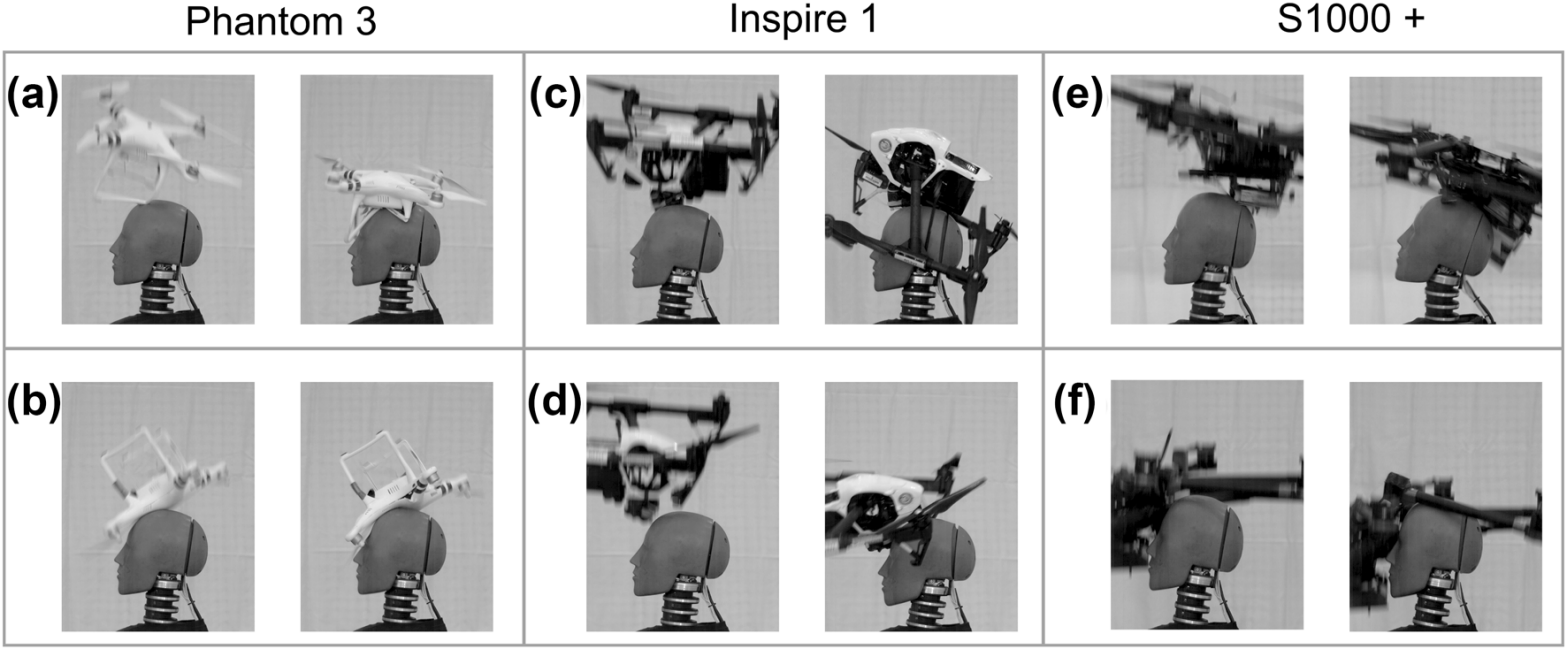
Representative falling impact tests. (a) The UAS leg struck the top of the head (left) and deformed (right). When the UAS body strikes the top of the head, velocity was already greatly reduced; (b) The UAS was dropped in an upturned position to assess a different impact orientation. Upon impact with the top of the head (left), the UAS body deforms (right); (c) The body of the UAS struck the top of the Hybrid III head (left). Upon impact, the arms swing down, but the overall body does not deform (right); (d) In a similar impact, the body/payload of the UAS struck the forehead of the head (left). No body deformation occurs (right) before the UAS falls away from the head; (e) The body/payload of the UAS struck the top of the head (left). The body deformed slightly before falling back away from the head (right); (f) One of the propeller arms struck the top of the head (left) and broke off from the UAS, which continued to fall away from the head (right).

**Figure 5:**
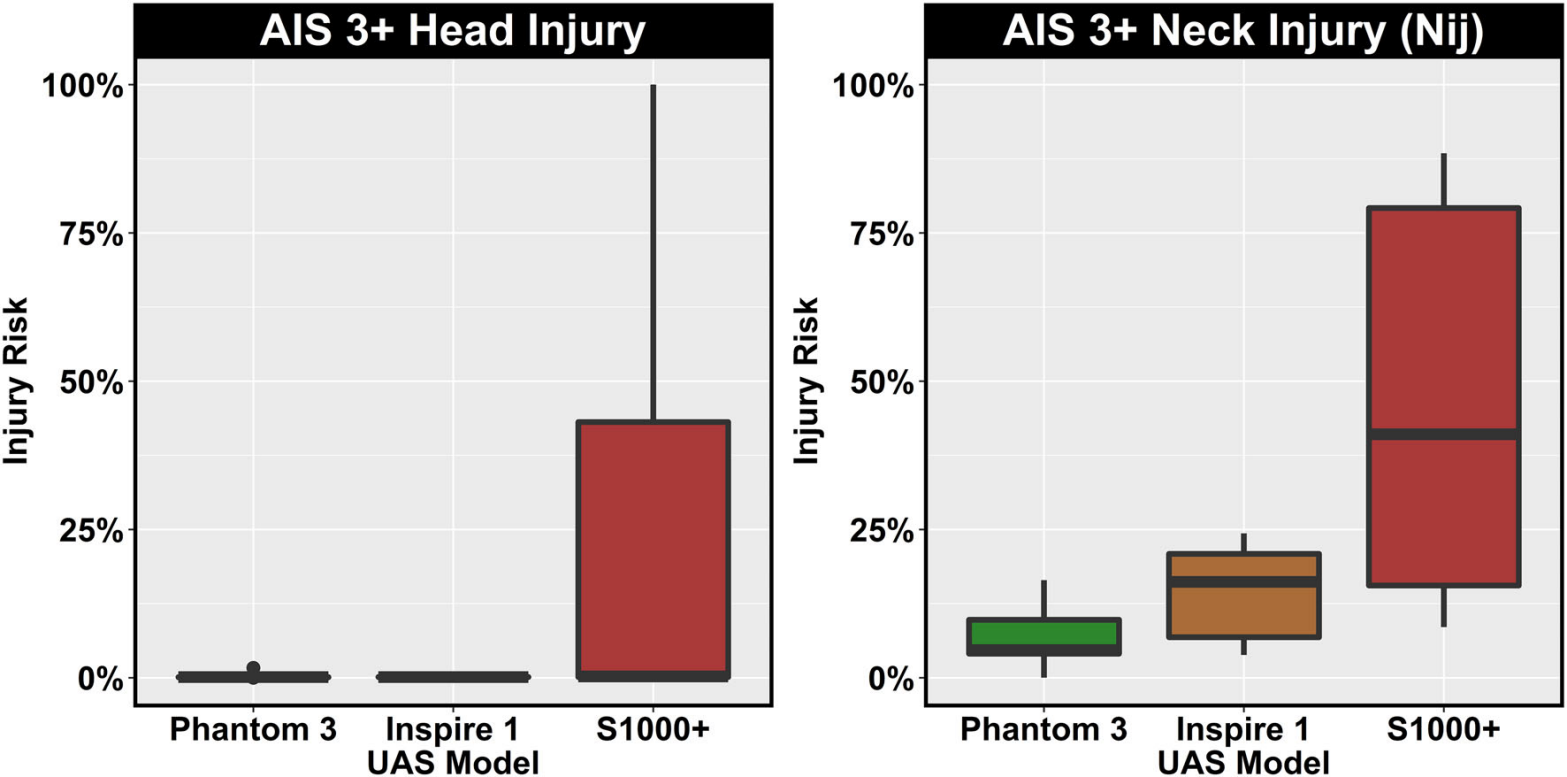
AIS 3+ head and neck injury risk. Head and neck injury risk were found to increase with increasing UAS mass. UAS orientation during impact varied considerably, leading to some tests estimating high likelihood of injury while others estimated no injury.

**Figure 6:**
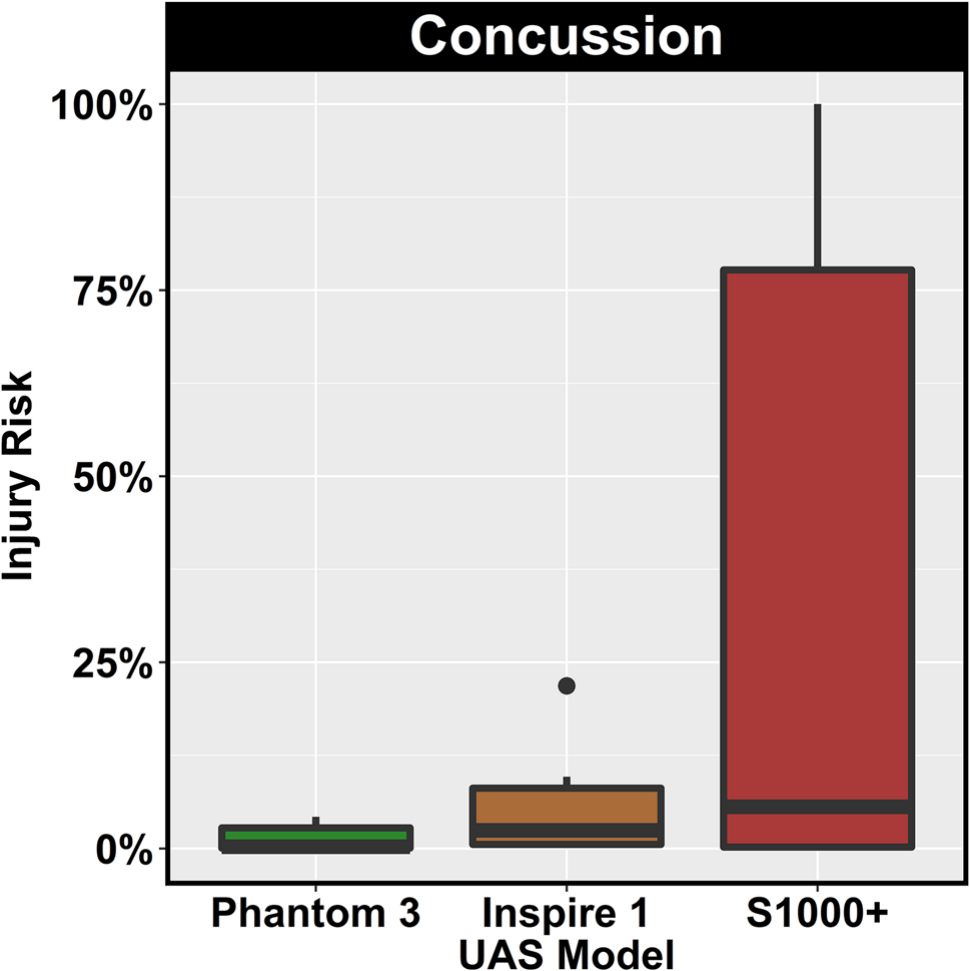
Summary of concussion risk by UAS model. Risk of concussion was found to increase with increasing UAS mass. Falling impact tests were more severe than live flight tests and were associated with higher levels of risk.

A total of 18 falling impact tests were conducted, 7 with DJI Phantom 3 (Figs. 4a and 4b), 6 with DJI Inspire 1 (Figs. 4c and 4d), and 5 with DJI S1000+ (Figs. 4e and 4f). In general, impacts from falling impact tests were of a higher severity than the live flight test impacts (Table 3). Impact orientations varied between the falling impact tests, resulting in a wide range of biomechanical parameters for each UAS model. The biomechanical parameters measured in this study increased with increasing mass. Further, the impact orientation with the Hybrid III varied from test to test. By testing several possible ways in which the UAS may fall and then impact a person’s head, a better estimate for the range injury risk could be determined.

**Table 3:** Biomechanical summary of falling impact tests.

	DJI phantom 3	DJI inspire 1	DJI S1000+
Peak linear acceleration (g)	32 (13–35)	62 (50–70)	86 (34–505)
Peak rotational acceleration (rad/s^2^)	3386 (1570–4966)	4268 (2924–5682)	5533 (3441–13,474)
Peak axial force (*N*)	1936 (623–2172)	3216 (2139–3537)	8029 (1883–10,219)
HIC_15_	12 (2–17)	28 (21–40)	186 (11–1591)
*N*_ij_	0.40 (0.23–0.48)	0.80 (0.76–0.96)	1.89 (0.93–2.52)

Increasing UAS mass was associated with higher severity impacts. Variation in impact orientation for each UAS resulted in a wide range of biomechanical values. Results are presented as Median (Interquartile Range)

### Injury Risk

Since biomechanical measurements were higher for falling impact tests than live flight tests, estimates for injury risk were also increased for falling impact tests. DJI Phantom 3 was still not associated with catastrophic head injury greater than 5%, while impacts from DJI S1000+ were estimated to result in wider ranges of injury, with some tests estimating 100% injury risk (Fig. 5). Impacts from DJI Inspire 1 were not likely to result in skull fracture. For all of the live flight impact tests, the highest risk of injury was AIS 3+ neck injury as estimated by *N*_ij_. For the four successful tests, these risk values ranged from 3.9 to 11.6%, with the 11.6% risk stemming from the 3rd impact with the DJI Phantom 3. Risk of neck injury due to impact from DJI Phantom 3 was below 10% during falling impact tests, while injury risks of 70% represented the median due to falling impacts from DJI S1000+. Impacts from the DJI Inspire 1 resulted in appreciable neck injury risk estimations (Fig. 5).

Concussion risk during live flight tests was estimated to be less than 5%, while the falling impact tests resulted in a wider range of concussion risks. Several impacts from the DJI S1000+ estimated 100% risk of concussion (Fig. 6). The higher levels of concussive risk relative to AIS 3+ head injury risk stem from concussion being a lower severity injury.

## Discussion

UAS regulations have progressed over the last several years to allow for increased operations; however, flight over people is still not allowed given the lack of knowledge regarding potential risk to humans from UAS impacts. This research represents a first step towards estimating risk of head and neck injury by conducting impact tests into a Hybrid III dummy through live flight and falling impact test configurations. The risk functions employed in this study come from motor vehicle occupant and football impact data but represent a good initial estimate to inform research geared towards future regulations.

A greater number of falling impact tests than live flight impact tests were conducted in this study. Impacting the head of the Hybrid III test dummy with an operational UAS proved to be a challenging task, which is why no successful flights were conducted with DJI Inspire 1. Further, the center of mass during these live flight tests was not always aligned with the Hybrid III head during impact. UAS leg or arm impacts transfer less energy from the UAS to the Hybrid III upon impact, leading to lower overall risk estimates (Table 2). In these impacts, the center of mass of the UAS is either redirected away from the headform or is not aligned with the headform during impact. Beyond this, the UAS models tested deformed upon impact, dissipating some of the overall impact energy towards deformation rather than transfer to the Hybrid III. The arm of the DJI S1000+ that struck the head broke off upon impact, while the leg of the DJI Phantom 3 deformed before deflecting away from the Hybrid III head (Figs. 2 and 3). The initial non-centric impact, coupled with the deformation of the UAS, likely contributed towards the overall lower risk values observed in live flight testing. Redirection away from the head and deformation of the UAS after impact limit the energy transfer to the head and are key to the safe design of an UAS suitable for flight over people. The variation in impact orientation and mass distribution demonstrates the need for comprehensive testing to fully characterize the risks associated with UAS impacts.

During falling impact tests, a variety of UAS impact orientations were investigated. Direct impacts from the base of the UAS, as well as indirect impacts from arms or legs of the UAS, were tested. For the indirect impacts, similar deformation patterns to live flight tests were noted on high speed video, as the impacting arm broke off or the impacting leg deformed before redirecting the UAS away from the head. Falling impact tests generally resulted in higher energy impacts than live flight tests despite lower impact velocities. Though these were free fall tests from a height of 5.5 m, the falling impact tests were more controlled than the live flight tests. Direct contact between the base of the UAS, where much of the overall mass is concentrated, and the Hybrid III head was attained in several tests. For these tests, overall risk estimates were increased (Figs. 5 and 6), with some of the tests estimating a high likelihood of injury.

While redirection away from the head was observed in most tests, it is still possible that the center of mass of the UAS could align with the head during impact. The 3^rd^ live flight test with the DJI Phantom 3 represented an impact in which the center of mass was aligned with the head during impact and resulted in higher biomechanical parameters than other live flight tests (Table 2). While this test likely represented the worst-case scenario for a live flight impact from the DJI Phantom 3, a similar test was not able to be conducted with the DJI Inspire 1 or S1000+. The successful impact with the DJI S1000+, in which an arm struck the Hybrid III head before breaking off, likely represented the best case scenario for this UAS model. The challenges in controlling impact conditions for these live flight impacts highlight the need for a modified experimental approach, such as a guided flight test rig to test UAS impact configurations.

For the DJI Inspire 1 and S1000+ , wide ranges in injury risk were observed for the falling impact tests (Figs. 5 and 6). The levels of risk generally varied based on the orientation of the UAS. Impacts in which the center of mass of the UAS, which is non-deformable in these models, was aligned with the head during impact were associated with higher risk than impacts with the deformable arms or legs of the UAS. Most of the DJI Phantom 3 is deformable, which explains the low levels of risk estimated for those impacts. The data from the tests in this study suggest that UAS deformation and deflection away from the head result in less severe impacts with lower estimates of injury risk.

The current UAS regulations restrict the max speed of UAS to about 45 m/s and the max mass to 25 kg. These values are more than twice as large as either the top speed or mass of the three UAS models used in this study. Given that many of the falling impact tests resulted in estimated risk of injury over 50%, further consideration to the maximum mass threshold should be taken if UAS are to be permitted to operate over people. While these tests only provide estimates of risk, the variety of impact configurations tested in this study and the appreciable risk values highlight the potential for catastrophic injury from UAS-person interaction.

It has been reported widely that the 50th percentile Hybrid III neck is longitudinally stiffer than the human neck. Sances *et al*. reported that the Hybrid III transmits about 75% of applied force to the lower neck, compared to only about 25% for cadavers.^24^ The Hybrid III neck has limited compliance in axial loading, which would be expected to produce greater force measurements and head accelerations than would be obtained with humans. It is difficult to assess how much lower the measured forces in this study should be to be more biofidelic, as cadaveric studies rarely record upper neck load measurements. Similarly, there is not a way to relate head acceleration measurements to human measurements. Because of these unknowns, the measured values could not be adjusted based on a transfer function between the Hybrid III response and cadaveric response. Though the Hybrid III dummy is imperfect as a human surrogate, it is the most widely available anthropomorphic test device for estimating risk of injury and has been used in a variety of traumatic impact loading events. Currently, no anthropomorphic test device exists specifically for assessing injury risk due to impacts from UAS.

This study was limited in investigating only three available UAS, but the different masses of the UAS tested provided a continuum from which injury estimates using intermediate masses may be extrapolated. The number of live flight tests was also limited in this study. Though the falling impact tests allowed for some improvement in impact orientation over live flight tests, none of the tests in this study were controlled. We recommend the use of a guided rig in future tests in order to accurately control the orientation of the UAS upon impact. N_ij_ likely overestimates low end risk, but is currently used in safety standards for automotive applications (FMVSS and NCAP). The underlying data that generated the concussion risk curve in this study comes from the Head Impact Telemetry System (HITS), which has been associated with errors for individual acceleration measurements. Compared to reference values from a Hybrid III head, acceleration measurements for individual impacts have been observed to vary by as much as 40%, though these large discrepancies have been found for impacts to the facemask in which the accelerometers of HITS may become decoupled from the headform.^4^,^12^ The effect of these errors is minimized when looking at the overall distribution of data, as the concussion risk function does.^7^ For concussive impacts, accelerations measured by HITS were similar to those reported in the National Football League dataset, which did not consider low magnitude, non-injurious head impacts.^18^ The HITS-based risk function represents the best risk function currently available as it considered both concussive and non-concussive impacts. Lastly, risk functions do not currently exist for UAS-person interactions, so the risk functions utilized in this study only represent estimates for injury risk.

Three commercially-available UAS were tested in two distinct testing environments to estimate the risk of head and neck injury to humans. By testing a range of masses and different impact configurations between the UAS and the Hybrid III, a better estimate for the levels of injury risk was determined. Designs that deform upon impact or redirect the UAS away from the head transfer less energy and resulted in lower severity impacts. Impacts with the smallest UAS tested in this study, the DJI Phantom 3, were associated with catastrophic injury risks below 5%. Some impacts from the two larger UAS investigated in this study estimated a high likelihood of injury and design alterations are likely necessary prior to these UAS being permitted to operate over people. More controlled and robust testing in the future will work to completely capture the level of risk associated with UAS-person interaction. These injury risk data represent a necessary foundation for the development of future UAS regulations on operations over people.
